# Homeopathy for cancer?

**DOI:** 10.3747/co.2007.128

**Published:** 2007-08

**Authors:** E. Ernst

**Keywords:** Homeopathy, cancer, complementary medicine, CAM

## INTRODUCTION

Homeopathy is “a therapeutic method of using preparations of substances whose effects when administered to healthy subjects correspond to the manifestation of the disorder in the individual patient”.^1^. The discipline was developed by Samuel Hahnemann (1755–1843) about 200 years ago. Hahnemann postulated that

- if a remedy causes a symptom in a healthy volunteer, then it can be used to treat that symptom in a patient (the “like cures like” principle).
- if a remedy is potentized (that is, diluted and sucussed), it becomes more rather than less effective (the “memory of water” theory).
- all diseases originate from the “itch” (psora), gonorrhea (sycosis), or syphilis (lues).

The third of these assumptions is now all but forgotten (I suspect that homeopaths feel embarrassed by its overt incorrectness), but despite the fact that the two other axioms also fly in the face of science, they still form the basis of homeopathy today.

Initially Hahnemann was remarkably successful, and homeopathy conquered much of the world.^2^. With hindsight, this early popularity probably accrued because, unlike many other therapies of the time, homeopathy was not outright harmful. With the eventual emergence of conventional treatments that generated more good than harm, homeopathy’s popularity faded.

## OBSERVATIONAL DATA

Today homeopathy is back with a vengeance. Why? Nobody really knows. The reasons are probably complex.^3^. One contributing factor seems to be that observational studies regularly show that patients receiving homeopathic treatments experience benefit.^4^,^5^. Others insist that any treatment that has been around for 200 years has “stood the test of time.”

Experience, the “test of time,” and observational studies all have one thing in common: the lack of a control. To be able to draw conclusions about cause and effect, a positive or negative control is needed. Observational data are, by definition, uncontrolled and unreliable. Causal inferences are therefore not appropriate.

Of course, medicine has a long tradition of disregarding this rather obvious fact.^6^. Whenever doctors administer a treatment to a patient outside of a clinical trial (that is, in an uncontrolled fashion), they are likely to attribute the ensuing outcome to the specific effects of their intervention. In other words, practitioners regularly make causal inferences on less than solid grounds.

**FIGURE 1:**
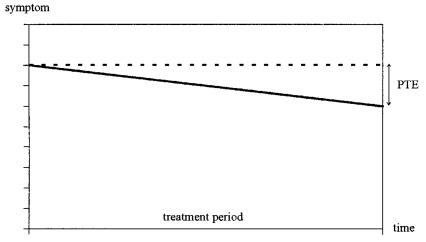
Schematic analysis of a typical treatment situation. PTE = perceived therapeutic effect.

It would be constructive to create conceptual clarity about what really is going on in such a situation. Figure 1 schematically depicts the case of a patient (or a group of patients) receiving homeopathy. Over time, symptoms improve, and a therapeutic effect is therefore perceived. The assumption of homeopaths is, therefore, that this “perceived therapeutic effect” is attributable to the specific effects of their intervention.

**FIGURE 2:**
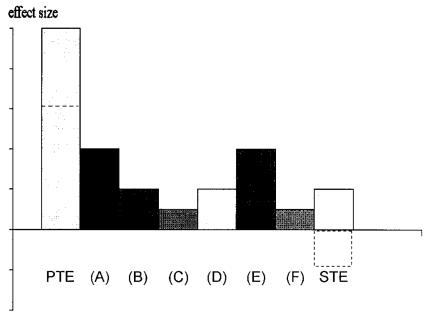
Schematic differentiation of factors contributing to the perceived therapeutic effect (PTE). (A) Natural course of the disease; (B) concomitant treatments, for example, self-administered over-the-counter drugs that patients forget to tell their doctors about; (C) regression toward the mean; (D) Hawthorn effect; (E) therapist–patient interaction; (F) social desirability, that is, patients saying that they are improved to please their doctor. The specific therapeutic effect (STE) is, for example, the pharmacologic action of a drug. A negative STE (dashed line) reduces the size of the PTE (dashed line), but does not necessarily abolish it totally.

In reality, the “perceived therapeutic effect” can be caused by a multitude of effects^7^. Figure 2 shows schematically the range of factors that could be involved. It is easy to see that, even if the specific therapeutic effect were to be negative (that is, a homeopathic treatment is harmful), the total perceived therapeutic effect could still be positive. It follows that ineffective (and even harmful) interventions can be falsely associated with overall improvement.

## RIGOROUSLY CONTROLLED STUDIES

To minimize the effects of confounding and bias, only one choice is available: to conduct rigorously designed randomized clinical trials (rcts). Some homeopaths insist that such studies cannot be done or are meaningless in homeopathy. The same homeopaths usually cheer whenever a rct emerges which suggests that homeopathy is efficacious. The fact that such trials exist clearly demonstrates that rcts^8^. of homeopathy are possible.

In the area of cancer, only very few rcts of homeopathy have been published—just five, in fact.^9^.

Kulkarni 10 conducted a RCT to test the effectiveness of homeopathy on the severity of radiotherapy-related side effects. Patients with different types of cancer (*n* = 82) were randomized into three parallel arms receiving either placebo, cobaltum C30, or causticum C30 (the “C” means centesimal potency). Patients were evaluated weekly using an 18-point radiation reaction profile, and the average grading was calculated at the end of the study. Compared with placebo, the reaction profile was lower in both experimental groups.

Oberbaum.^11^ tested the effectiveness of Traumeel S (TRS, New York, NY, U.S.A.) for chemotherapy-induced stomatitis after allogeneic or autologous stem-cell transplantation. Patients (*n* = 30) were randomised to two groups: the Traumeel S oral rinse or a placebo rinse. Traumeel S contains arnica 2X, calendula 2X, millefolium 3X, chamomilla 3X, symphytum 6X, belladonna 2X ana 0.1 mL, aconitum 2X 0.06 mL, bellis perennis 2X 0.05 mL, hypericum 2X 0.03 mL, echinacea angustifolia 2X, echinacea purpurea 2X ana 0.025 mL, hamamelis 1X 0.01 mL, mercurius sol. 6X 0.05 g, and hepar sulfuris 6X 0.1 g (the “X” means decimal potency). Significant differences favouring the Traumeel S group were observed in terms of reduction in the severity or duration (or both) of stomatitis and in time to worsening of symptoms. Patients in that group showed a reduction in oral pain and discomfort, in dryness of mouth and tongue, in difficulty of swallowing, and in dysphagia.

Balzarini.^12^ tested the effectiveness of homeopathic treatment for skin reactions during radiotherapy treatment for breast cancer. Patients (*n* = 61) were randomized into a group receiving three granules of belladonna 7CH twice daily and X-ray 15CH once daily (the “CH” means centesimal Hahnemannian potency) or a group receiving placebo. Patients treated with homeopathy noted less hyperpigmentation and a decrease in skin temperature, but these differences were no longer significant by the end of the 10-week follow-up. Total severity scores favoured homeopathy, but statistical significance for the difference was noticed only during recovery.

Jacobs and colleagues.^13^ evaluated homeopathy for menopausal symptoms in 83 breast cancer survivors. Patients who suffered from an average of 3 hot flushes daily for a month before the trial were randomized into three groups: a placebo combination and a verum single remedy; a verum combination medicine and a verum single remedy; and two placebo combinations. Single remedies consisted of 35 different homeopathic medications, mainly sepia, calcarea carbonica, sulphur, lachesis, and kali carbonicum (mostly high potencies). The combination remedy was “Hyland’s menopause,” which contains amyl nitrate, sanguinaria canadensis, and lachesis. No significant differences were found between the three groups in terms of symptom score over a 1-year period. A significant improvement in general health score was observed in both homeopathy groups as compared with the placebo group. A significant increase in headache was observed in the combination homeopathy group.

Thompson *et al.*^14^ compared homeopathy with placebo in 53 breast cancer survivors with estrogen withdrawal symptoms. Patients randomized to homeopathy were individually prescribed 71 different remedies, most commonly sulphur, sepia, carcinosin, natrum muriaticum, belladonna, and arnica (mostly high potencies). No significant differences between the experimental and the placebo group were noted.

## COMMENT

Homeopathy is again popular—also with cancer patients. Observational data suggesting effectiveness have to be interpreted with great caution. Randomized controlled trials are scarce, and those currently available are burdened with significant methodologic limitations.^9^. All of the existing RCTs are in the realm of cancer palliation and supportive care. Independent replication of these data are not currently available.

Few experts would argue that low-potency homeopathic remedies (preparations that contain pharmacologically active molecules) may generate clinical effects. The dispute centres mainly on the issue of whether high potency remedies (preparations diluted beyond the Avogadro number) can be effective. Potencies (dilutions) of botanic substances beyond 7C (meaning 7 dilutions, each 1:100) do not contain a sufficiently significant number of molecules of the original material to be pharmacologically active.^1^.

Considering the biologic implausibility of high-potency homeopathy, my conclusions have to be conservative. There is no evidence at all that homeopathic remedies can change the natural history of any cancer. The few RCTs of homeopathy are in the realm of cancer palliation and supportive care and have not generated convincing evidence of a beneficial effect. For indications other than cancer, the evidence from rigorous RCTs is also not convincing^15^. As a result, there is no reason to believe that homeopathic medicines have anything to offer to patients suffering from cancer or other conditions apart from non-specific effects. However, to generate the placebo effect, we do not necessarily need placebos.
